# Value of lipoprotein(a) in predicting severity of coronary artery stenosis in patients with chronic coronary artery disease: a cross-sectional study in Vietnam

**DOI:** 10.3389/fcvm.2025.1669234

**Published:** 2025-11-13

**Authors:** Hao Thai Phan, Mai Thi Tuyet Ho

**Affiliations:** 1 Internal Medicine Department, Pham Ngoc Thach University of Medicine, Ho Chi Minh, Vietnam; 2 Tam Anh Hospital, Ho Chi Minh, Vietnam

**Keywords:** lipoprotein (a) [Lp (a)], gensini coronary score, predicting, coronary artery disease, coronary stenosis

## Abstract

**Introduction:**

Coronary artery disease (CAD) remains a leading cause of morbidity and mortality worldwide. Lipoprotein (a) [Lp(a)] has emerged as an independent risk factor for CAD, but its role in predicting coronary severity in Vietnamese populations remains unclear.

**Objectives:**

To evaluate the value of Lp(a) in predicting the severity of coronary artery stenosis in chronic CAD.

**Materials and methods:**

This cross-sectional study was conducted at Tam Anh General Hospital from June 2024 to June 2025, including 138 patients diagnosed with chronic CAD. Demographic, clinical, laboratory, and coronary angiographic data were collected. CAD severity was assessed using the Gensini score. Logistic regression and ROC analysis were employed to evaluate the predicting value of Lp(a).

**Results:**

Severe CAD (Gensini score >40) was present in 31.9% of the cohort. Patients with Lp(a) ≥30 mg/dL exhibited a significantly higher prevalence of severe CAD (72.5% vs. 8.0%). Lp(a) levels correlated strongly with the Gensini score. The optimal cut-off for predicting severe CAD was 30.6 mg/dL (AUC = 0.869). Multivariate analysis confirmed Lp(a) as an independent predictor.

**Conclusions:**

Lp(a) ≥30 mg/dL is strongly associated with severe coronary artery stenosis. Lp(a) is a valuable independent predictor of CAD severity and may serve as an essential tool for risk stratification in clinical practice.

## Introduction

Coronary artery disease (CAD) is a major global health concern and a leading cause of morbidity and mortality worldwide (1–3), particularly in low- and developing countries. Vietnam is witnessing a growing burden of CAD due to rapid economic development and changing lifestyles. Traditional risk factors such as hypertension, diabetes, dyslipidemia, smoking, and sedentary behavior are prevalent, but novel markers like Lipoprotein (a) [Lp(a)] are emerging as significant contributors to cardiovascular risk (4–6).

Lp(a) is structurally similar to low-density lipoprotein (LDL) but possesses an additional apolipoprotein (a) component, enhancing its pro-atherogenic and pro-thrombotic properties (5, 7–11). Elevated Lp(a) levels have been consistently linked to increased risks of myocardial infarction, stroke, and peripheral artery disease. Despite substantial evidence from Western populations, data regarding the role of Lp(a) in Southeast Asian and specifically Vietnamese populations remain limited (12–15).

This study aimed to investigate the association between Lp(a) levels and CAD severity in Vietnamese patients with chronic CAD using the Gensini scoring system, providing region-specific insights that may inform clinical practice.

## Materials and methods

This was a cross-sectional study conducted at Tam Anh General Hospital, Ho Chi Minh City, Vietnam, between June 2024 and June 2025. The study population included patients aged 18 years or older with chronic coronary artery disease (CAD) diagnosed according to the 2019 ESC guidelines, who underwent invasive coronary angiography and had available Lipoprotein(a) [Lp(a)] measurements. Patients with congenital coronary anomalies, end-stage renal disease, active malignancy, pregnancy, or breastfeeding were excluded. Consecutive eligible patients during the study period were enrolled to minimize selection bias. Demographic, clinical, and laboratory characteristics—including comorbidities, lipid profiles, and Lp(a) levels—were collected, along with angiographic findings. The primary outcome was the association between Lp(a) concentration and CAD severity, which was assessed using the Gensini score. Lp(a) was measured by immunoturbidimetric assay (Beckman Coulter AU680 analyzer). Statistical analyses included descriptive statistics, Mann–Whitney *U* test, chi-square test, Spearman correlation, receiver operating characteristic (ROC) analysis, and logistic regression modeling. A significance level of *p* < 0.05 was considered statistically significant.

## Results

### Study population

**Figure 1:**
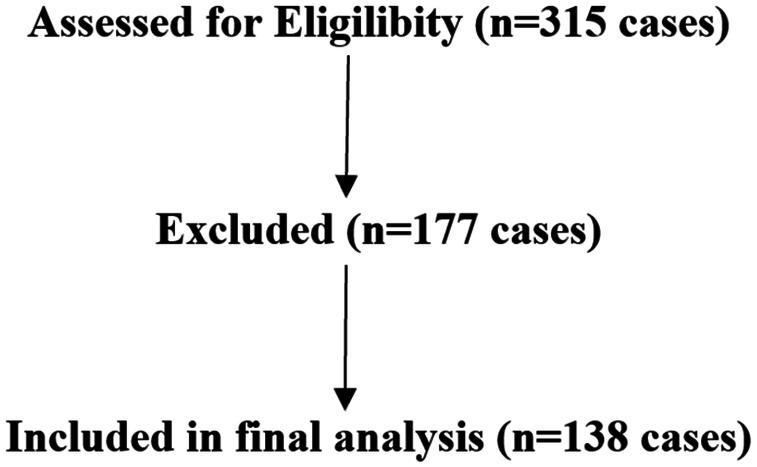
Study flowchart.

A total of 315 patients were analyzed. There were 177 patients were excluded included: stage V CKD (*n* = 42), active malignancy (*n* = 31), congenital anomalies (*n* = 7), pregnancy/breastfeeding (*n* = 3), no Lp(a) data (*n* = 94) (Figure 1).

### Demographics and clinical characteristics

The mean age of the study population was 68 ± 12 years, and 74 patients (54%) were male.The baseline demographic and cardiovascular risk factors of the two groups are summarized in Table 1. There were no significant differences between the groups in age, gender distribution, body mass index (BMI), type 2 diabetes mellitus, dyslipidemia, or smoking status between patients with Lipoprotein (a) [Lp(a)] levels ≥30 mg/dL and those with Lp(a) levels <30 mg/dL. However, hypertension was significantly more prevalent in the high Lp(a) group (96.2% vs. 85.0%, *p* = 0.032).

**Table 1:** Demographic characteristics by Lp(a) level.

Characteristics	Lp(a) < 30 mg/dL (*n* = 86)	Lp(a) ≥ 30 mg/dL (*n* = 52)	*p*-value
Age (years), mean ± SD	68 ± 12	68 ± 12	0.883
Male, *n* (%)	44 (51.2%)	30 (57.7%)	0.447
BMI (kg/m²), median (IQR)	23.2 (21.8–25.8)	23.8 (22.3–25.5)	0.368
Hypertension, *n* (%)	74 (85.0%)	50 (96.2%)	0.032
Dyslipidemia, *n* (%)	79 (91.0%)	48 (94.1%)	0.713
Type 2 Diabetes, *n* (%)	40 (46.0%)	28 (55.0%)	0.403
Smoking, *n* (%)	25 (29.0%)	15 (29.4%)	1.000

The clinical and laboratory characteristics are presented in Table 2. There were no significant differences between groups regarding heart rate, systolic and diastolic blood pressure, platelet count, creatinine, blood glucose, HbA1c, HDL-C, triglycerides, NT-proBNP, or Troponin Ths levels (all *p* > 0.05).

**Table 2:** Clinical and laboratory characteristics.

Characteristics	Lp(a) < 30 mg/dL	Lp(a) ≥ 30 mg/dL	*p*-value
Heart rate (bpm), median (IQR)	74 (64–87)	75 (65–83)	0.970
Diastolic BP (mmHg), mean ± SD	76 ± 13	75 ± 11	0.627
Systolic BP (mmHg), median (IQR)	128 (117–141)	132 (117–142)	0.577
Hemoglobin (g/dL), mean ± SD	13.7 ± 1.8	12.9 ± 1.8	0.012
Platelets (G/L), median (IQR)	240 (198–287)	251 (213–291)	0.359
Troponin Ths (pg/mL), median (IQR)	13.9 (7.7–23.6)	13.4 (7.1–27.5)	0.956
NT-proBNP (pg/mL), median (IQR)	163 (64.1–998)	117 (55.2–431)	0.346
Creatinine (µmol/L), mean ± SD	76 ± 21	82 ± 25	0.149
Glucose (mmol/L), median (IQR)	6.5 (5.6–7.6)	6.7 (5.6–8.3)	0.566
HbA1c (%), median (IQR)	6.0 (5.8–6.8)	6.2 (5.7–7.6)	0.350
LDL-C (mmol/L), median (IQR)	2.0 (1.7–2.7)	3.3 (2.1–3.9)	<0.001
HDL-C (mmol/L), median (IQR)	1.1 (0.9–1.3)	1.1 (0.9–1.3)	0.778
Total Cholesterol (mmol/L), median (IQR)	3.6 (3.1–4.8)	5.1 (3.6–5.7)	<0.001
Triglycerides (mmol/L), median (IQR)	1.6 (1.0–2.4)	1.4 (1.1–2.5)	0.553

However, patients with Lp(a) ≥ 30 mg/dL had significantly:

- Lower hemoglobin levels: 12.9 ± 1.8 g/dL vs. 13.7 ± 1.8 g/dL; *p* = 0.012
- Higher LDL-C levels: 3.3 mmol/L vs. 2.0 mmol/L; *p* < 0.001
- Higher total cholesterol levels: 5.1 mmol/L vs. 3.6 mmol/L; *p* < 0.001

The median Gensini score was significantly higher in the Lp(a) ≥ 30 mg/dL group compared to the <30 mg/dL group [57 (IQR: 38–84) vs. 11 (IQR: 3–26); *p* < 0.001]. Severe multi-vessel disease (≥3 vessels with >50% stenosis) was observed in 43.1% of the Lp(a) ≥ 30 mg/dL group vs. 10.3% in the <30 mg/dl group (*p* < 0.001) (Table 3).

**Table 3:** Coronary angiography findings.

Characteristics	Lp(a) < 30 mg/dL	Lp(a) ≥ 30 mg/dL	*p*-value
Gensini Score, median (IQR)	11 (3–26)	57 (38–84)	<0.001
No significant stenosis, *n* (%)	35 (40.2%)	0 (0.0%)	<0.001
One-vessel stenosis, *n* (%)	26 (29.9%)	14 (27.5%)	0.847
Two-vessel stenosis, *n* (%)	17 (19.5%)	15 (29.4%)	0.213
Three-vessel stenosis, *n* (%)	9 (10.3%)	22 (43.1%)	<0.001

### Comparison by CAD severity

When stratified by Gensini score, there were no significant differences in demographic characteristics or most clinical and laboratory parameters between the mild and severe stenosis groups, except that patients with severe stenosis had significantly lower hemoglobin (12.9 vs. 13.6 g/dL; *p* = 0.026) and higher LDL-C (3.0 vs. 2.1 mmol/L; *p* = 0.012) and total cholesterol levels (4.8 vs. 3.7 mmol/L; *p* = 0.040). Lp(a) levels were significantly higher in the severe group (69 vs. 12 mg/dL; *p* < 0.001) (Tables 4, 5).

**Table 4:** Demographics by CAD severity.

Characteristics	Gensini ≤ 40 (*n* = 94)	Gensini > 40 (*n* = 44)	*p*-value
Age (years), mean ± SD	69 ± 12	67 ± 13	0.532
Male, *n* (%)	49 (52.1%)	25 (56.8%)	0.740
BMI (kg/m^2^), median (IQR)	23.2 (21.8–25.7)	23.6 (22.2–25.7)	0.452
Hypertension, *n* (%)	81 (86.2%)	43 (97.7%)	0.073
Type 2 Diabetes, *n* (%)	42 (44.7%)	26 (59.1%)	0.163
Dyslipidemia, *n* (%)	86 (91.0%)	41 (93%)	0.996
Smoking, *n* (%)	27 (29.0%)	13 (30%)	1.000

**Table 5:** Clinical and laboratory characteristics by CAD severity.

Characteristics	Gensini ≤ 40 (*n* = 94)	Gensini > 40 (*n* = 44)	*p*-value
Heart rate (bpm), median (IQR)	74 (63–85)	75 (65–91)	0.322
Diastolic BP (mmHg), mean ± SD	75 ± 12	76 ± 11	0.715
Systolic BP (mmHg), median (IQR)	129 (118–141)	133 (116–145)	0.368
Ejection Fraction (%), median (IQR)	60 (56–65)	60 (56–64)	0.982
Hemoglobin (g/dL), mean ± SD	13.6 ± 1.8	12.9 ± 1.8	0.026
Platelets (G/L), median (IQR)	240 (201–279)	260 (213–302)	0.175
Creatinine (µmol/L), mean ± SD	77 ± 20	81 ± 27	0.451
LDL-C (mmol/L), median (IQR)	2.1 (1.7–3.2)	3.0 (1.9–3.7)	0.012
Total Cholesterol (mmol/L), median (IQR)	3.7 (3.1–5.1)	4.8 (3.4–5.6)	0.040
Lp(a) (mg/dL), median (IQR)	12 (7–23)	69 (43–106)	<0.001
Log Lp(a), median (IQR)	2.6 (2.0–3.2)	4.3 (3.8–4.7)	<0.001

### Distribution of Lp(a)

**Figure 2:**
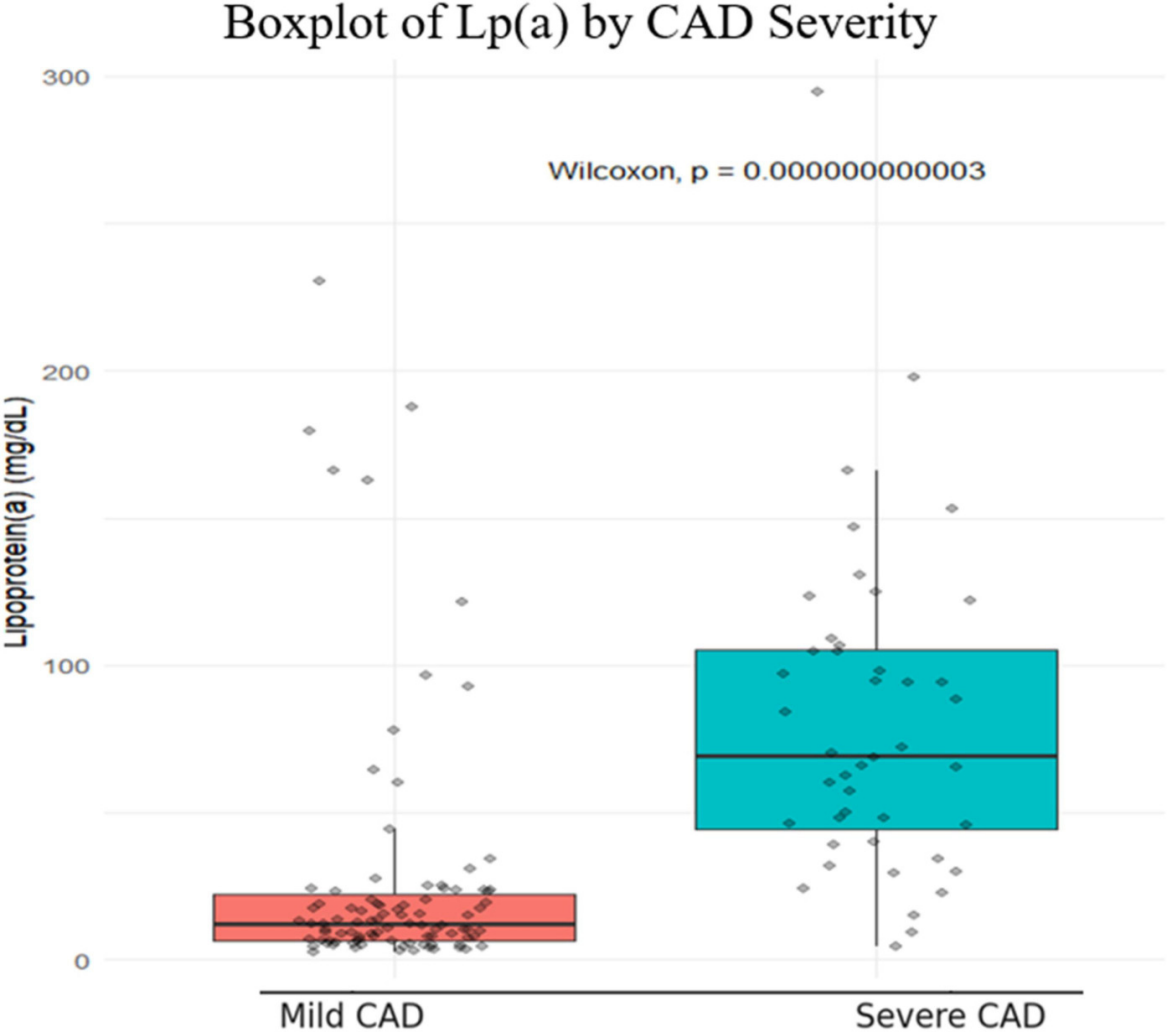
Boxplot of Lp(a) by CAD severity.

The boxplot showed that Lp(a) levels in the severe CAD group (Gensini > 40) were about six times higher than in the mild group (median 69 vs. 12 mg/dL), with a highly significant difference (*p* < 0.001) (Figure 2).

**Figure 3:**
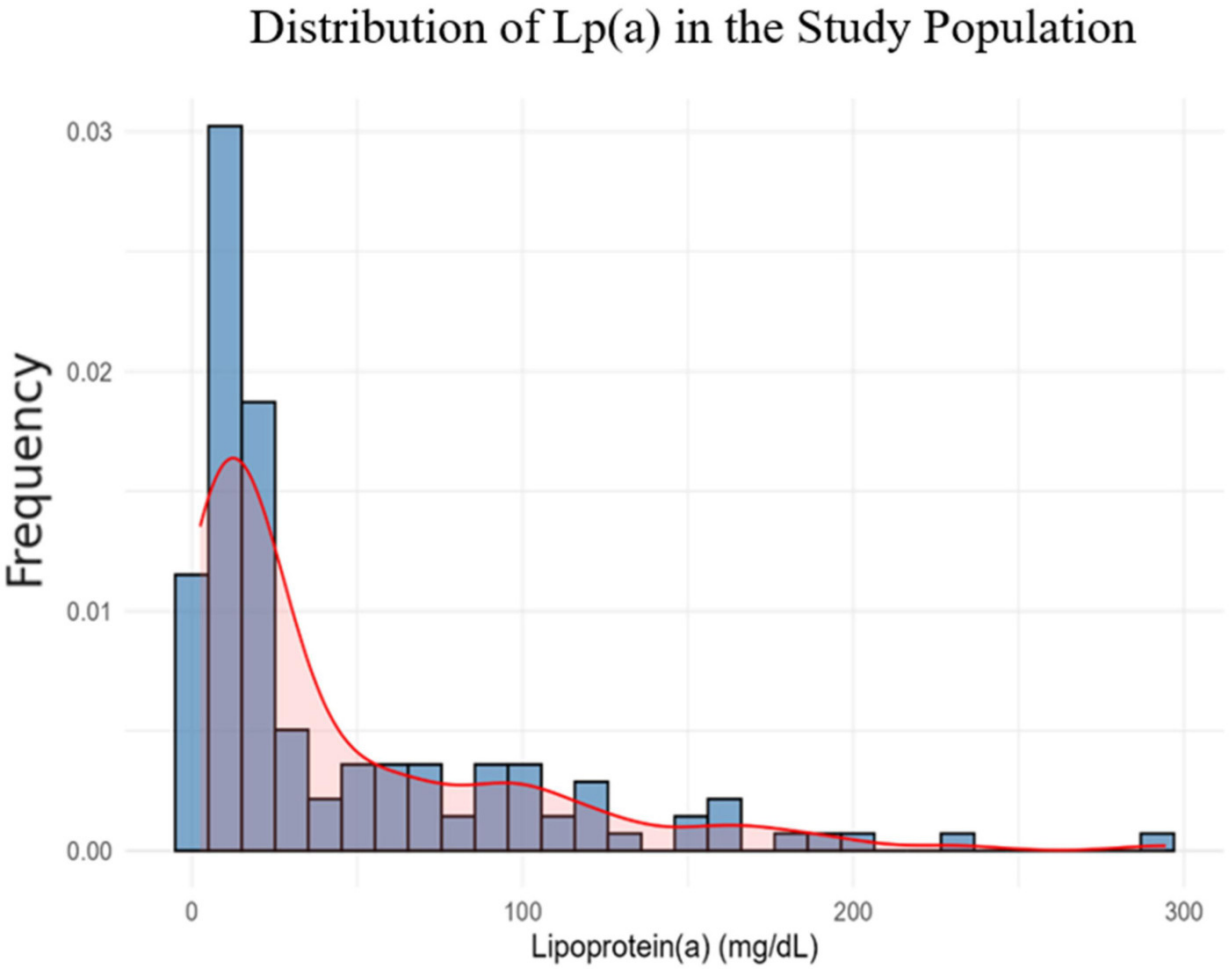
Lp(a) distribution in the study population.

The distribution of Lp(a) was right-skewed (*p* < 0.001), with most values below 30 mg/dL. Therefore, we applied a natural logarithmic transformation to normalize the data and improve the reliability of subsequent statistical analyses (Figure 3).

### Proportion of severe CAD by Lp(a) group

**Figure 4:**
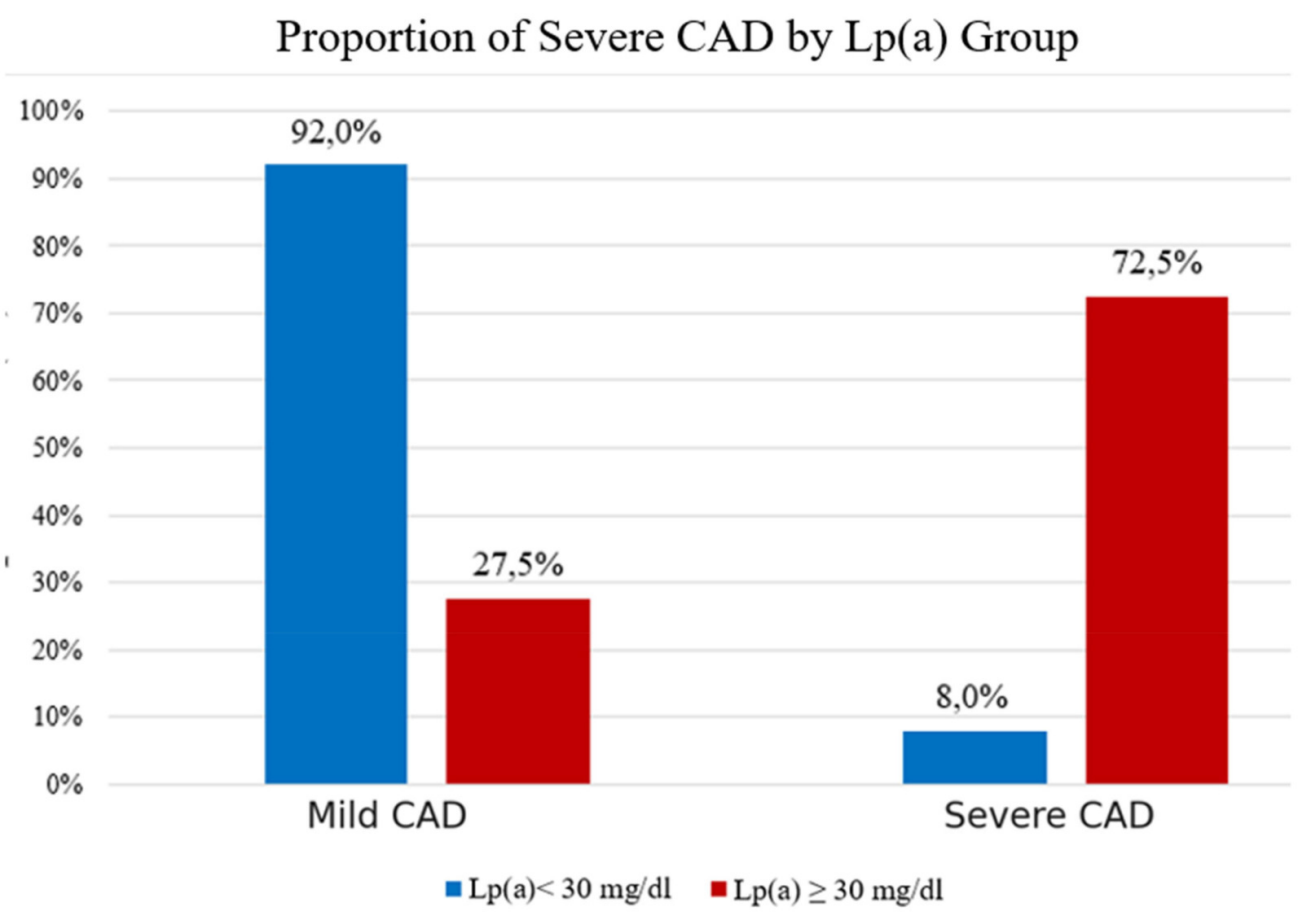
Proportion of severe CAD by Lp(a) group.

The study showed that the prevalence of severe coronary stenosis was significantly higher in the Lp(a) ≥ 30 mg/dL group (72.5%) compared to the < 30 mg/dL group (8.0%) (*p* < 0.001). Conversely, mild stenosis was more common in the low Lp(a) group (92.0%). These findings confirm that elevated Lp(a) is a strong risk factor for severe coronary artery disease in this population (Figure 4).

### Correlation between Lp(a) and Gensini score

**Figure 5:**
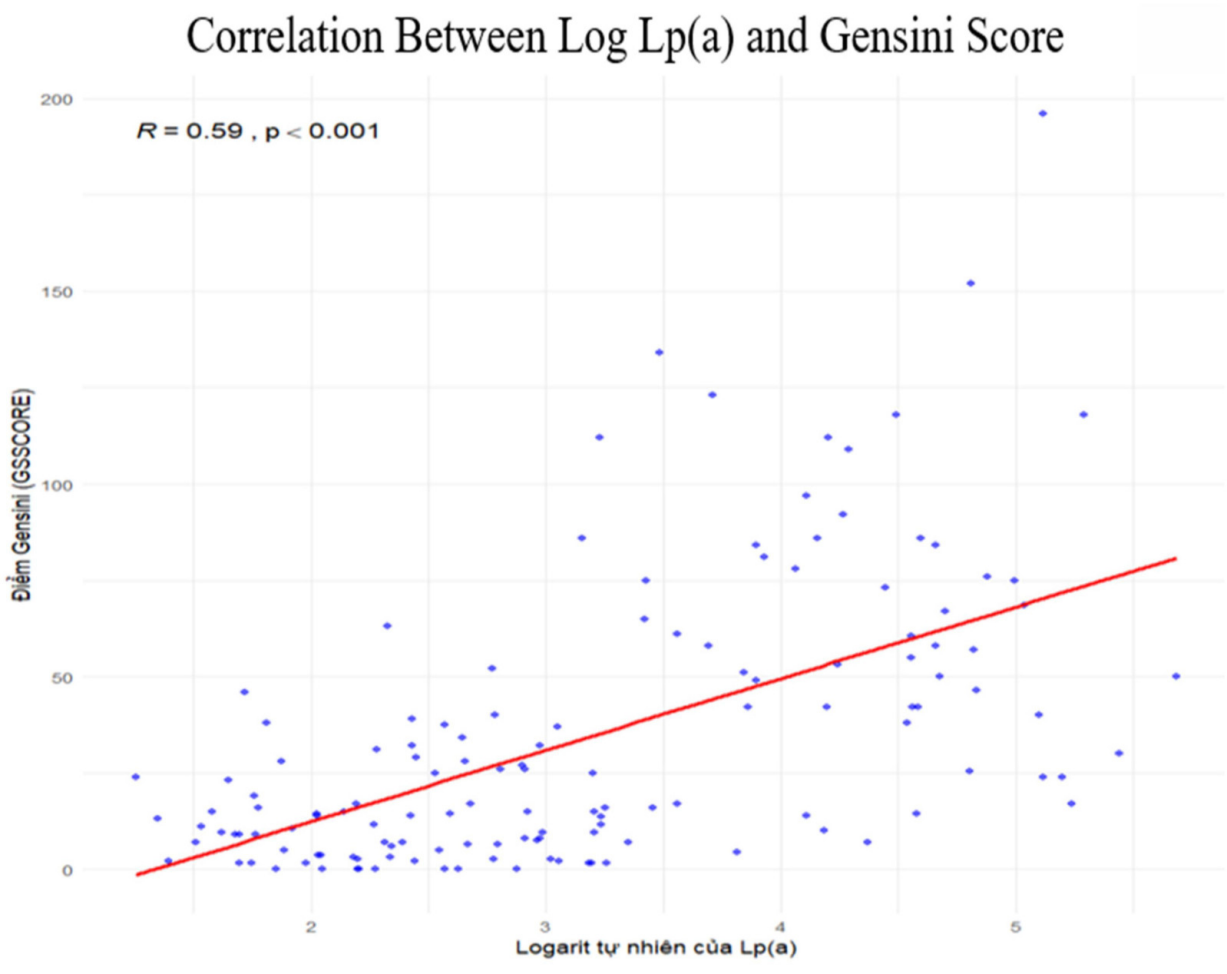
Correlation between Log Lp(a) and gensini score.

Spearman correlation analysis demonstrated a significant positive correlation between the natural logarithm of Lp(a) and the Gensini score (*ρ* = 0.59; *p* < 0.001). Higher Lp(a) levels were associated with more severe coronary stenosis, with a clear upward trend across the sample. No major outliers or abnormal distributions were observed, indicating a consistent relationship (Figure 5).

### ROC curve analysis

**Figure 6:**
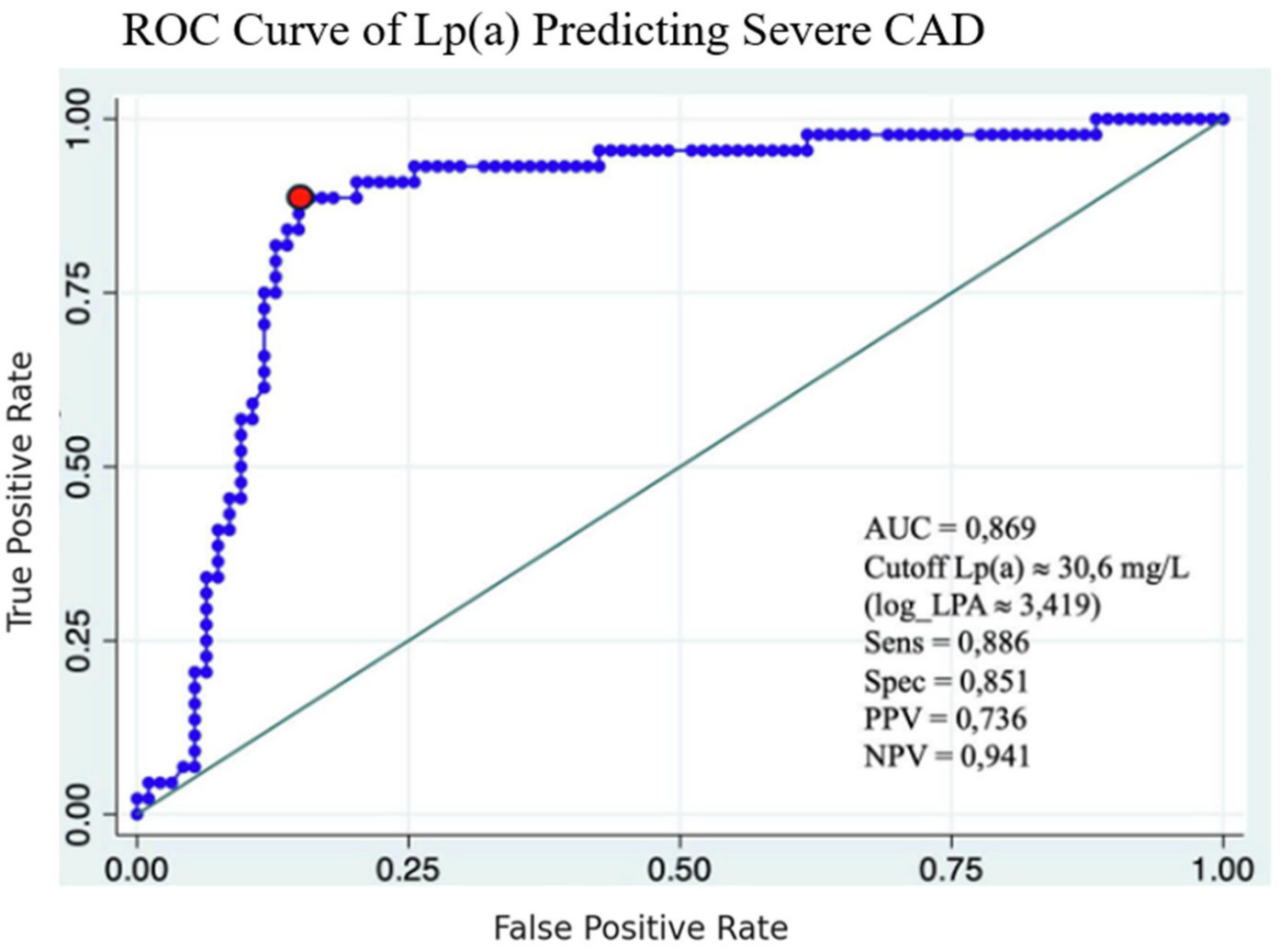
ROC curve of Lp(a) predicting severe CAD.

The ROC analysis identified 30.6 mg/dL as the optimal cutoff for Lp(a) to predict severe coronary stenosis, with a sensitivity of 88.6% and specificity of 85.1%. The positive and negative predictive values at this cutoff were 73.6% and 94.1%, respectively, highlighting the clinical value of Lp(a) in assessing disease severity (Figure 6).

### Logistic regression results

Univariate logistic regression showed that the natural logarithm of Lp(a) was strongly associated with severe coronary stenosis (OR = 4.55; 95% CI: 2.9–7.8; *p* < 0.001), with LDL-C (OR = 1.52; *p* = 0.012) and hemoglobin (OR = 0.79; *p* = 0.027) also showing significant associations. Lp(a) had the strongest predictive value. Hypertension and diabetes showed a trend but were not statistically significant. Other factors, including age, sex, smoking, dyslipidemia, BMI, and triglycerides, were not associated with disease severity. The wide confidence interval for hypertension suggests possible estimation bias due to limited sample size (Table 6).

**Table 6:** Univariate logistic regression.

Variable	OR	95% CI	*p*-value
Natural logarithm of Lp(a)	4.55	2.9–7.8	<0.001
Age	0.99	0.96–1.02	0.511
Female	0.83	0.4–1.7	0.607
Hypertension	6.90	1.3–127	0.067
Diabetes	1.79	0.9–3.7	0.116
Smoking	1.04	0.5–2.3	0.921
Dyslipidemia	1.27	0.4–6.0	0.733
Body Mass Index (BMI)	1.02	0.9–1.1	0.709
LDL-Cholesterol (mg/dL)	1.52	1.1–2.1	0.012
Hemoglobin (g/dL)	0.79	0.6–1.0	0.027
Triglycerides (mg/dL)	0.94	0.7–1.2	0.640

When performing multivariate logistic regression analysis, we selected variables with a *p*-value < 0.2 from the univariate logistic regression (including LDL-C, hemoglobin, hypertension, and diabetes). In the multivariate logistic regression, only the natural logarithm of Lipoprotein (a) [Lp(a)] remained a strong independent predictor of severe coronary stenosis (adjusted OR = 4.34, 95% CI: 2.6–8.0; *p* < 0.001). Although LDL-C and hemoglobin were significant in the univariate analysis, they lost significance after adjustment. Hypertension and diabetes were not independent predictors in either model (Table 7).

**Table 7:** Multivariate logistic regression.

Variable	Adjusted OR	95% CI	*p*-value
Natural logarithm of Lp(a)	4.34	2.6–8.0	<0.001
LDL-Cholesterol	1.05	0.7–1.6	0.829
Hemoglobin	0.93	0.7–1.2	0.565
Hypertension	2.34	0.3–49.4	0.471
Diabetes	2.15	0.8–5.9	0.121

## Discussion

This cross-sectional study of 138 patients with chronic coronary artery disease (CAD) demonstrated a strong association between elevated Lipoprotein(a) [Lp(a)] levels and the severity of coronary artery stenosis. Patients with Lp(a) ≥ 30 mg/dL were significantly more likely to have severe CAD (72.5%) compared to those with Lp(a) < 30 mg/dL (8.0%). Among various factors analyzed, only the natural logarithm of Lp(a) remained an independent predictor of severe stenosis in both univariate and multivariate logistic regression, with an adjusted odds ratio of 4.34 (95% CI: 2.6–8.0, *p* < 0.001). These findings emphasize the strong predictive value of Lp(a) in assessing anatomical CAD burden. Among the 138 study participants, we recorded 74 males and 64 females, with males accounting for 53.6% (*n* = 74). This proportion is lower than that reported in international studies by David Leistner et al. (10) and Christoph Sinning et al. (16), where the male proportions were 70.1% and 77.4%, respectively, in European countries. In a 2024 study conducted in China by Yang et al. (17), the proportion of males was comparable to that in our study, accounting for 58.28%. In contrast, a domestic study by Thái Thị Phương Thảo et al. (18) reported a higher proportion of males (72.9%) compared to our findings. Overall, coronary artery disease (CAD) tends to develop earlier in men than in women (6, 19). In our study, the male-to-female ratio was nearly equal, reflecting a shifting epidemiological trend of CAD in the population of Ho Chi Minh City. When analyzing BMI data based on weight and height, we recorded a median BMI of 23.3 kg/m^2^ in our study, with an interquartile range of 22.1–25.7 kg/m^2^. The study conducted by Krzysztof Dyrbuś et al. (20), primarily involving a Turkish population, reported a significantly higher median BMI compared to ours, with a median value of 28.1 kg/m² (interquartile range 25.1–31.8 kg/m²). Regarding biochemical characteristics, our study recorded a mean serum creatinine concentration of 78 ± 23 µmol/L in the study population. In the group with lipoprotein(a) < 30 mg/dL, the mean creatinine level was 76.15 ± 20.79 µmol/L, while in the group with Lp(a) ≥ 30 mg/dL, it was 82.20 ± 25.06 µmol/L. There was no statistically significant difference between the two groups (*p* = 0.149). Our findings are consistent with those reported by He et al. (21).

Diagnostic performance of Lp(a) was further evaluated using receiver operating characteristic (ROC) analysis. An optimal cut-off value of 30.6 mg/dL yielded excellent results: an area under the curve (AUC) of 0.869, with sensitivity of 88.6% and specificity of 85.1%. These figures indicate a high discriminatory capacity for identifying patients with severe coronary stenosis. Interestingly, the AUC in our study was higher than that reported in several Western studies, suggesting possible ethnic or genetic influences on Lp(a) expression and cardiovascular risk profiles in Asian populations.

When compared to the international literature, our results are in agreement with major studies that also highlight the independent role of Lp(a) in coronary disease severity. For instance, Wang et al. found a similar association in a large cohort of patients with myocardial infarction (8), and the European LipidCardio study confirmed the value of Lp(a) using both Gensini and SYNTAX scores (10). Notably, our identified threshold (30.6 mg/dL) closely aligns with the ≥30 mg/dL level proposed by the European Atherosclerosis Society (EAS) as a clinically relevant cutoff (16, 22). These concordant findings add weight to current guideline recommendations that recognize Lp(a) as an important, genetically determined cardiovascular risk factor that warrants measurement in certain high-risk populations. Pare et al. reported that there is diversity in concentration and isoform distribution among different ethnic groups, and that the risk of cardiovascular events increases with higher lipoprotein(a) levels, especially in South Asian populations (23). A recent study by Cesaro et al. (24), conducted within the RELACS study on 975 European patients, also confirmed the association between elevated Lp(a) levels and the complexity of coronary artery disease as assessed by the SYNTAX-I and Gensini scores. Patients with Lp(a) ≥150 nmol/L had a significantly higher median Gensini score compared to those with lower levels (16.0 vs. 10.0; *p* < 0.01).

Despite its strengths, the study has limitations. The relatively small sample size may limit the generalizability of the results. Additionally, imbalances in subgroup distributions, particularly for traditional risk factors like hypertension or diabetes, may affect the precision of odds ratio estimates. Furthermore, as a cross-sectional study, it lacks longitudinal follow-up data to assess the impact of elevated Lp(a) on clinical outcomes such as myocardial infarction, revascularization, or cardiovascular mortality.

Nonetheless, the findings of this study offer important clinical implications. First, Lp(a) measurement may serve as a valuable tool for risk stratification in patients with chronic CAD, particularly in those with well-controlled LDL-C levels but persistent residual cardiovascular risk. Second, identifying patients with high Lp(a) could prompt consideration of more aggressive risk-reduction strategies, including lipoprotein apheresis, lifestyle modification, and future Lp(a)-lowering therapies currently in development. However, to our knowledge, this is the first study in Vietnam to systematically evaluate the association between Lp(a) and CAD severity using the Gensini score. Ethnic and genetic differences may influence Lp(a) expression and its impact on cardiovascular risk. Previous studies Enkhmaa et al. (25) and Leistner et al. (10) have shown significant ethnic variation in Lp(a)-related cardiovascular risk. Therefore, our findings provide region-specific insights that complement existing international evidence and highlight the need for further studies in Southeast Asian populations.

Lipoprotein(a) [Lp(a)] is structurally similar to low-density lipoprotein (LDL), but it possesses an additional apolipoprotein(a) component that confers unique biological properties. Apolipoprotein(a) is covalently bound to apolipoprotein B-100, and its structural resemblance to plasminogen contributes to impaired fibrinolysis, thereby promoting thrombosis. Moreover, Lp(a) carries oxidized phospholipids, which exert potent pro-inflammatory and pro-atherogenic effects. These mechanisms collectively accelerate atherosclerotic plaque development, progression, and instability, increasing the risk of myocardial infarction and other adverse cardiovascular events.

In addition to these biological mechanisms, ethnic variability in Lp(a) expression is increasingly recognized as an important determinant of cardiovascular risk. Studies have demonstrated that Lp(a) levels and their impact on atherosclerotic disease differ substantially across populations, with Southeast Asian cohorts showing distinct distributions compared with Western counterparts. This variability may be related to genetic factors, including differences in kringle IV type 2 repeat polymorphisms, as well as environmental influences. Our findings, therefore, provide region-specific insights into the role of Lp(a) in Vietnamese patients, complementing global evidence and underscoring the importance of context-specific data in cardiovascular risk assessment.

In our multivariate logistic regression, we adjusted for key covariates including LDL-C, hemoglobin, hypertension, and diabetes. Despite these adjustments, we acknowledge that residual confounding cannot be fully excluded. Important unmeasured factors such as systemic inflammation, variability in renal function, or socioeconomic determinants may still influence the observed association between Lp(a) and coronary artery disease severity. This limitation highlights the need for larger, multicenter studies with more comprehensive adjustment for potential confounders to validate and extend our findings in broader and more diverse Vietnamese populations.

In conclusion, this study supports existing international evidence and current guidelines that recognize Lp(a) as an important biomarker of atherosclerotic burden. With its strong association with severe coronary stenosis and high diagnostic performance, Lp(a) measurement should be considered in routine cardiovascular risk assessment, particularly in populations where traditional risk factors do not fully explain disease severity.

## Conclusion

In conclusion, Lp(a) is a strong and independent predictor of severe coronary artery stenosis in patients with chronic coronary artery disease. A cut-off value of 30.6 mg/dL provides high sensitivity and specificity for identifying high-risk patients. These results support the clinical relevance of routine Lp(a) assessment in cardiovascular risk evaluation and may guide more personalized prevention and treatment strategies (Central Illusion).

**Central Illustration:**
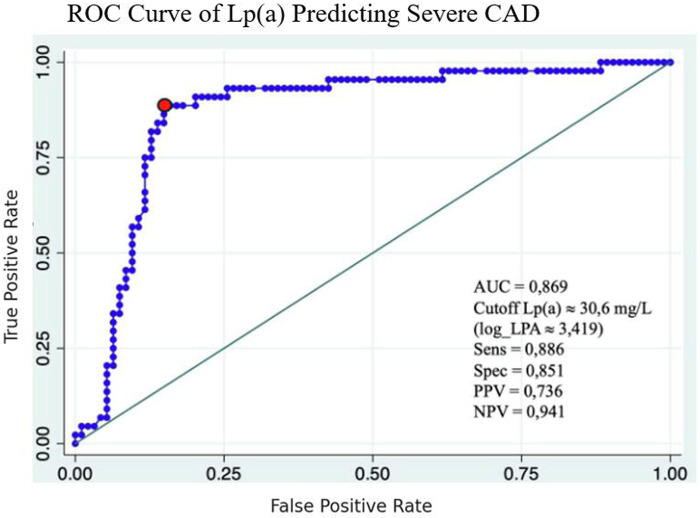
ROC Curve with AUC = 0.869, Lp(a) cutoff 30.6 mg/dL, sensitivity 88.6%, specificity 85.1%, correlation graph of log Lp(a) vs. Gensini score.

## Data Availability

The raw data supporting the conclusions of this article will be made available by the authors, without undue reservation.
